# Vulva Self-Image and Sexual Function After Female External Genital Plastic Surgery

**DOI:** 10.1007/s00266-024-04570-5

**Published:** 2024-12-09

**Authors:** Tatiana Turini, Geisa Sant Ana, Aline Mizusaki Imoto, Maria Eduarda Alves Martins

**Affiliations:** 1 Instituto de Cirurgia Plastica Tatiana Turini, SGAS II St. de Grandes Áreas Sul 616 Conjunto A Lote 116/117 Sala 1 - Asa Sul, Brasília, DF 70200001 Brazil; 2 Hospital Regional da Asa Norte, Brasilia, DF Brazil; 3 Escola Superior de Ciencias da Saude, Brasilia, DF Brazil; 4 Centro Universitario de Brasília, Brasilia, DF Brazil

**Keywords:** Female genital plastic surgery, Vulva, Nymphoplasty

## Abstract

**Introduction:**

Female external genital plastic surgery (vulvoplasty) is among the most performed aesthetic surgeries in Brazil. Vulva self-image can be defined as women's perception of their vulva and is usually related to sexual function. The objective of this study was to evaluate the impact and interrelationship of female external genital plastic surgery on self-image and sexual function.

**Method:**

This quantitative longitudinal descriptive study includes a retrospective analysis of 48 medical records from June 2017 to July 2021. The sexual function questionnaire (SFQ-28) and female genital self-image scale (FGSIS) answered by the patients in the preoperative and six postoperative month were analyzed.

**Result:**

The mean age of the operated patients was 36.25 years (range 18–59). The median FGSIS score was 13 in the preoperative and 27.5 in the six postoperative month, with a significant positive difference in all items. There was a significant positive difference in the following SFQ-28 domains: desire, arousal (cognitive), orgasm, enjoyment, and partner (*p* < 0.005)

**Conclusion:**

In our cohort, female external genital plastic surgery (vulvoplasty) elicited a positive relationship between genital self-image and sexual function, in addition to improving orgasm.

**Level of Evidence IV:**

This journal requires that authors assign a level of evidence to each article. For a full description of these Evidence-Based Medicine ratings, please refer to the Table of Contents or the online Instructions to Authors www.springer.com/00266.

## Introduction

Female external genital plastic surgery (vulvoplasty) is among the most performed aesthetic surgeries in Brazil.According to the most recent data released by the International Society of Aesthetic Plastic Surgery (ISAPS), 14,986,982 aesthetic plastic surgeries were performed worldwide in 2022. In the world ranking of surgical procedures, labiaplasty is in 18th place, with 1.3% of all procedures, and vaginal rejuvenation with 0.5% [1]. Brazil, in the ISAPS statistics from 2022 is the first country worldwide to perform plastic surgeries 2,049,257, (37,170 were labiaplasty, the country with the highest number of these surgeries worldwide [1].

Vulva self-image may be usually related to sexual function [2]. Vulva self-image can be defined as the perception that women have about their vulva. Women whose genital self-image is more positive have sexual behaviour such as going to the gynecologist annually, more frequent use of masturbation and sex toys [3], and usually more positive sexual function. These women are usually willing to accept new practices with their partner, feeling free, comfortable, and sexually satisfied [2]. There is a tendency for more mature women to have higher genital self-image [4]. Patients with this profile are less likely to consider the surgical procedure (labioplasty, vaginal rejuvenation, G-spot amplification, hymen restoration, clitoral hood modification, and mons pubis reduction). Therefore, women with negative self-image tend to have usually more sexual dysfunction [2, 5, 6].

Negative changes in self-image may be related to the search for the surgical procedure in most cases [7]. Genital self-image is a health aspect that must be understood by the professionals involved in the approach to this dysfunction; therefore, it is essential to know the factors that determine this negative perception of oneself. This promotes expanded access to information for better planning of actions to help these women, avoiding damage to self-esteem and mental health [5].

Body dysmorphic disorder may be observed in women seeking labiaplasty. It is characterized by a concern with a perceived defect that is unobservable or seems mild to others; however, the individual's concern is markedly excessive. These women, in turn, are vulnerable and can be compared and/or equated with other body image disorders (e.g., bulimia nervosa). All factors involved in this situation may be nonspecific, including teasing by negative comments about the general or specific physical appearance of their genitalia. This context may increase their body dissatisfaction, leading to depression and low self-esteem [8–10].

Self-esteem is an important factor in sexual functioning [2, 11]. According to Edmonds (2010), the construct of self-esteem has fulfilled a trajectory of a certain strangeness, starting from a recent origin as a non-existent word in the English language until the 19th century, becoming today a global concept linked to a specific set of rationality for social and psychotherapeutic purposes [12].

Regarding sexual intercourse, the problem of lubrication should be highlighted. Women who feel ashamed of the vulva region or pain during sexual intercourse tend to lubricate less due to tension, which can cause even more discomfort during sex and could cause relationship problems and a feeling to escape from these moments that, instead of being pleasurable, become stressful [13].

The aging process and congenital or hormonal issues influence the vulva modification. In practice, there is a difference between women who will complain and want to improve the vulva region while others do not [14–16]. Although there is a relationship between the size of the labia minora and dissatisfaction with their appearance, labia minora with less protrusion has higher values of the genital self-image [4]. There are women with labia minora within the published limits sizes and, even so, seek the procedure [8, 17].

Therefore, given this dilemma, it is important that the surgeon be cautious when it comes to surgery for "abnormal" vulvas and that the surgery is not performed after suggestion but after a thorough examination and alignment of complaints, expectations, and guidance on the risks involving the surgical procedure [15, 16, 18].

The objective of the study is to evaluate the impact and interrelationship of female external genital plastic surgery (vulvoplasty) on self-image and sexual function using the data obtained from the female genital self-image scale (FGSIS) and sexual function questionnaire (SFQ-28).

## Methods

### Inclusion and Exclusion Criteria

Forty-eight cis women patients were included in the study. The inclusion criteria were women (no transgender patients were included) over 18 years, admitted by the surgery outpatient clinic of the Hospital Regional Asa Norte-Unified Health System (HRAN-SUS) and a private clinic of plastic surgery, with hypertrophy of the labia minora type 1 (excess of the labia minora near the vaginal introitus), type II (labial hypertrophy extending to the upper-lateral region of the clitoral hood) and III (labial hypertrophy extending to the whole clitoral hood), according to the classification of Cunha et al. [19], associated or not with sagging of the labia majora and/or excess clitoral hood, and willing to undergo female genital plastic surgery.

Exclusion criteria were patients who lost follow-up after surgery, had no active sex life (less than one sexual activity in the last year), were illiterate, had lesions suggestive of vulvar cancer, active infection in the genitalia (patients not operated on until the infection was resolved), and incomplete or incorrect completion of the study questionnaire.

Labia majora lipofilling was performed in 16 patients, to improve sagging in this region, mainly due to aging.

## Questionnaires

The SFQ-28 is a self-reported measure of female sexual function and was developed to be multidimensional and patient-centered. The SFQ-28 addresses all aspects of the sexual response cycle (desire, arousal, orgasm) as well as pain, which is consistent with the criteria in the Diagnostic and Statistical Manual of Mental Disorders and the newly generated American Foundation for Urologic Disease definitions [20, 21].

**Fig 1:**
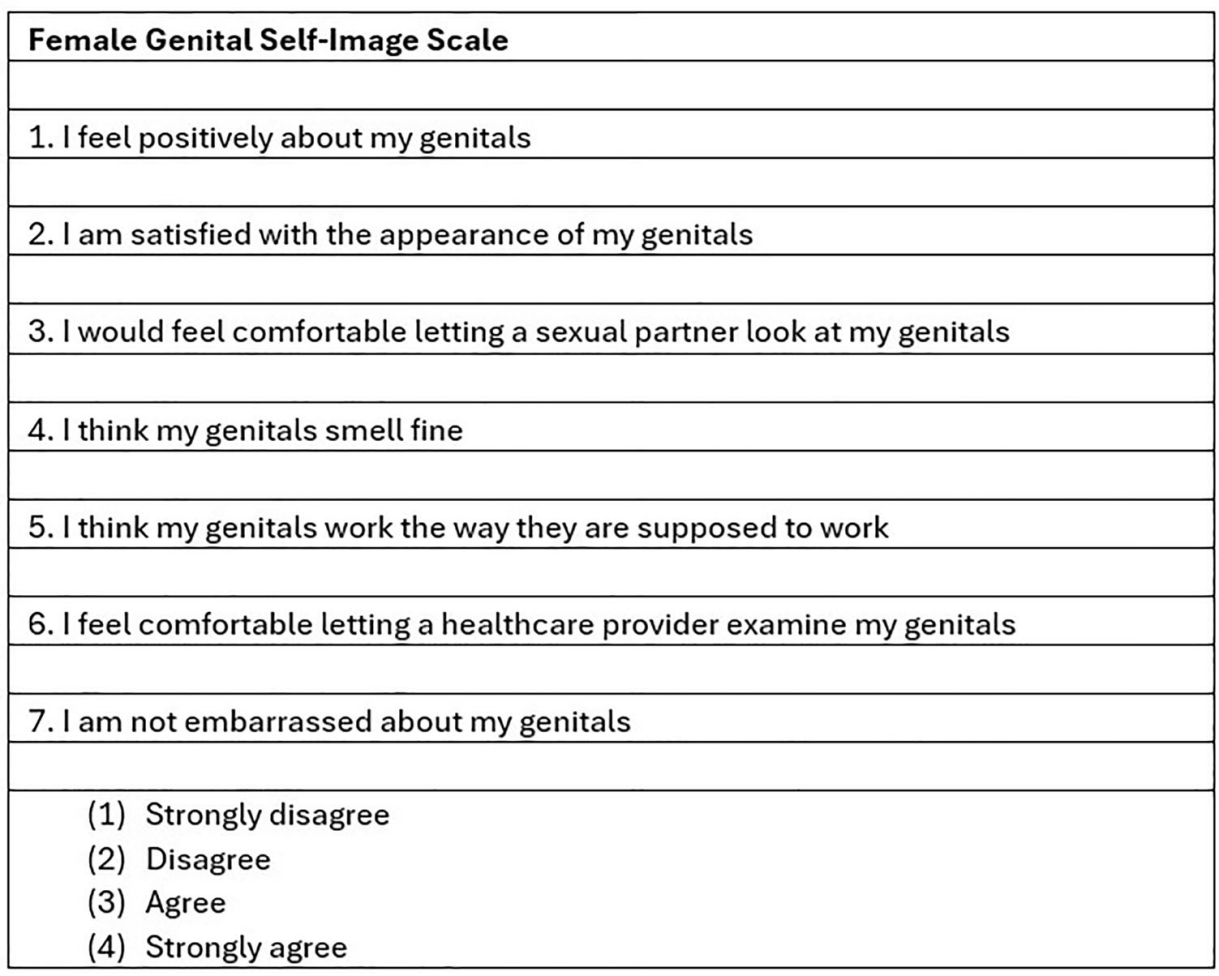
Female genital self-image scale questionnaire

Female Genital Self-image Scale (FGSIS) is a widely used and validated questionnaire translated into several languages, including Portuguese, that has established reliability, validity, and temporal stability. It measures the domains of desire, arousal, lubrication, pain, orgasm, and satisfaction and also results in a total score. Higher scores on each domain and the total score indicate more positive sexual function [2, 22–27] (Fig. 1).

## Data Collection

Data was collected between July 2017 and June 2021. The study was approved by the Institutional Review Board (IRB) under the number 3.253.314, which included the use of the SFQ-28 questionnaire. In the preoperative, all patients read and signed a standardized informed consent form that follows the guidance of the Brazilian Society of Plastic Surgery specific to "female genital plastic surgery" and the release of images and the SFQ-28 questionnaire for scientific purposes.

Data were collected from medical records to identify and analyze the sociodemographic profile, such as age, combined surgeries, type of anesthesia, postoperative complications, comorbidities, and techniques used in Excel® spreadsheets for later analysis.

The SFQ-28 and FGSIS answered by the patients in the preoperative and three and six postoperative months were analyzed along with follow-up photography.

## Data Analysis

The study was divided into descriptive, association, and correlation analyses. Data analysis was performed using the IBM Statistical Package for Social Sciences (SPSS) version 23 of 2015. The statistical significance level was defined as p-value ≤ 0.05.

The quantitative variables were initially evaluated for the normality of the data distribution by the Shapiro-Wilk test. Most of the null hypothesis of normality was rejected for most of the patients, which was also indicated in the analysis of the Q-Q plots. Considering the small sample size in several analyses, nonparametric tests were used: Wilcoxon test for related and independent samples; Mann-Whitney U test for comparison of two independent groups; Kruskal-Wallis test for comparison in variables with three or more categories; and Dunn's post hoc test for comparison between pairs when the Kruskal-Wallis test showed significant results.

## Surgical Technique

**Fig 2:**
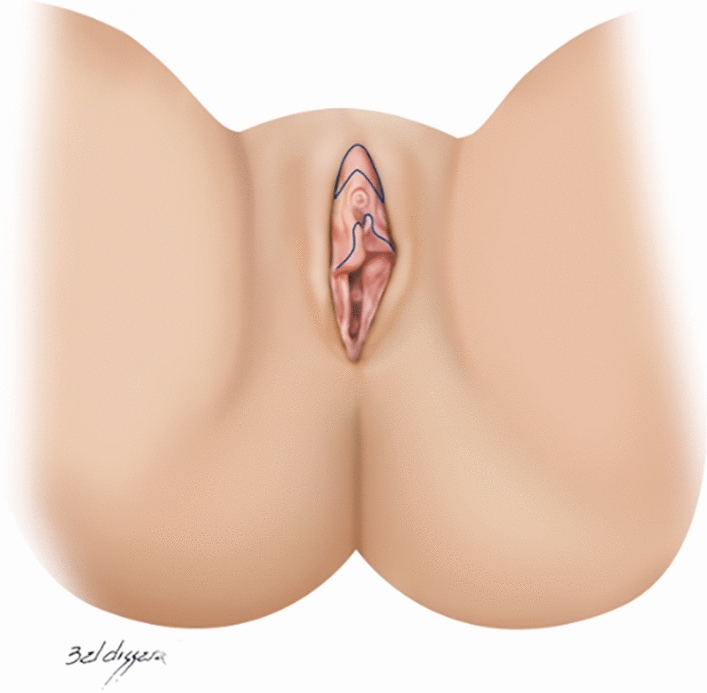
Clitoral hood and labia minora marking

**Fig 3:**
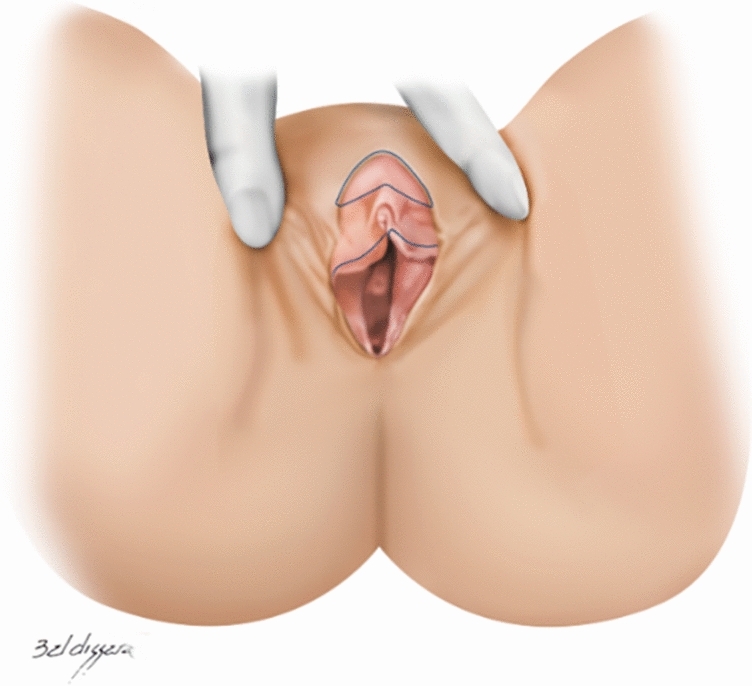
Clitoral hood and labia minora marking. Traction of region to show marking in frenulum region

The surgical technique performed was in "boomerang", which is the clitoral hood resection in its most cephalic portion combined with clitoripexy and labia minora resection longitudinally since most patients have the combination of labia minora and clitoral hood excess (Figs. 2 and 3) [28]. Labia majora lipofilling is associated with cases of sagging in this region.

## Results

The patient's age ranged from 18 to 59 years. Most patients (41.67%) were between 28 and 37 years, a mean of 36.25 years (+ 9.76%) and a median of 35.5 years (Table 1). More than half of the sample (64.58%) were from a public hospital-Unified Health System (SUS).

**Table 1:** Sociodemographic and surgical characteristics of patients submitted to female genital plastic surgery, Brasília, DF, 2022

	Item	n	%
Age range	18–27 years	8	16.67
	28–37 years	20	41.67
	38–47 years	14	29.17
	≥ 48 years	6	12.50
SUS / private clinic	SUS	31	64.58
	Private clinic	17	35.42
Excess labia minora resection	No	1	2.08
	Yes	47	97.92
Anesthesia	Local	39	81.25
	Peridural	6	12.50
	General	3	6.25
Refinements	No	42	87.5
	Yes	6	12.5
Complications	No	45	93.25
	Yes	03	6.25
Combined surgery	No	39	81.25
	Yes	9	18.75
Total		48	100.00

*SUS*: Public hospital-unified health system

In the procedures performed, local anesthesia was the most prevalent anesthesia used in 39 patients (81.25%). Epidural anesthesia was used in 12 patients (12.5%), and general anesthesia in three (6.25%). Anesthetic solution infiltration is used even in epidural and general anesthesia for better control of bleeding since the solution is 1:200,000 of adrenaline, especially in cases where only intimate surgery was performed, as shown in Table 1. Epidural and general anesthesias were used in combined surgeries, such as breast augmentation and liposuction, which are more common among procedures performed in conjunction with female genital plastic surgery.

As shown in Table 1, there were three cases of early complications (6.25%), two of hematoma of the labia minora and one of wound dehiscence. Three patients (6.25%) sought medical advice to undergo a secondary procedure due to late complications from surgeries performed by other surgeons.

Surgical refinement was performed on six patients. In one case, this was performed to improve a small asymmetry, through a small unilateral resection; in another case, there was also labia minora redundancy, in which a new tissue resection was performed; and in three cases, clitoral plication surgery (clitoripexy) was performed on patients who had previously had clitoral hypertrophy secondary to hormones. There were no cases of infection. Most of the patients never had surgery on the vulgar region before.

Table 2 shows that most patients were submitted to the "boomerang technique"; in only 3 cases, labioplasty was performed alone. Vulvoplasty as a whole comprised 41 cases (85.4%), in which resection of the labia minora, resection of the excess clitoral hood, and clitoripexy were combined. Among the 48 patients in the study, 16 were submitted to labia majora treatment to improve the sagging in this region, mainly due to aging.

**Table 2:** Operated genital areas of the patients included in the study, Brasília, DF, 2022

Female genital plastic surgery		
Female genital areas	n	%
Labia minora (Nymphoplasty/labioplasty)	**3**	**6.28**
Labia minora + labia majora	1	2.08
Labia minora + hood + clitoris	**41**	**85.4**
Labia minora + hood	1	2.08
Labia minora + posterior vaginal furcula	1	2.08
Hood + clitoris	1	2.08
Total	48	100

As provided in the methodology, the FGSIS and SFQ-28 questionnaires were answered spontaneously at the surgeon's invitation and in the service's routine, in the immediate preoperative and the three and six postoperative month period. All participants were invited and given the questionnaires at this moment. However, data from three postoperative month were not included in the study due to the low number of responders. One of the reasons for this loss is that some patients had consultation after one month and then returned only after six months, excluding the three postoperative month consultation. Another reason was a group of patients did not answer the questionnaire. There were three unidentified questionnaires, which were discarded.

Among the 48 patients in the study, 29 answered the FGSIS in the preoperative and 27 in the six postoperative month period, although 18 patients answered both questionnaires. Patients who failed to respond at some point did not enter the analysis. Incomplete questionnaires were also excluded.

**Fig 4:**
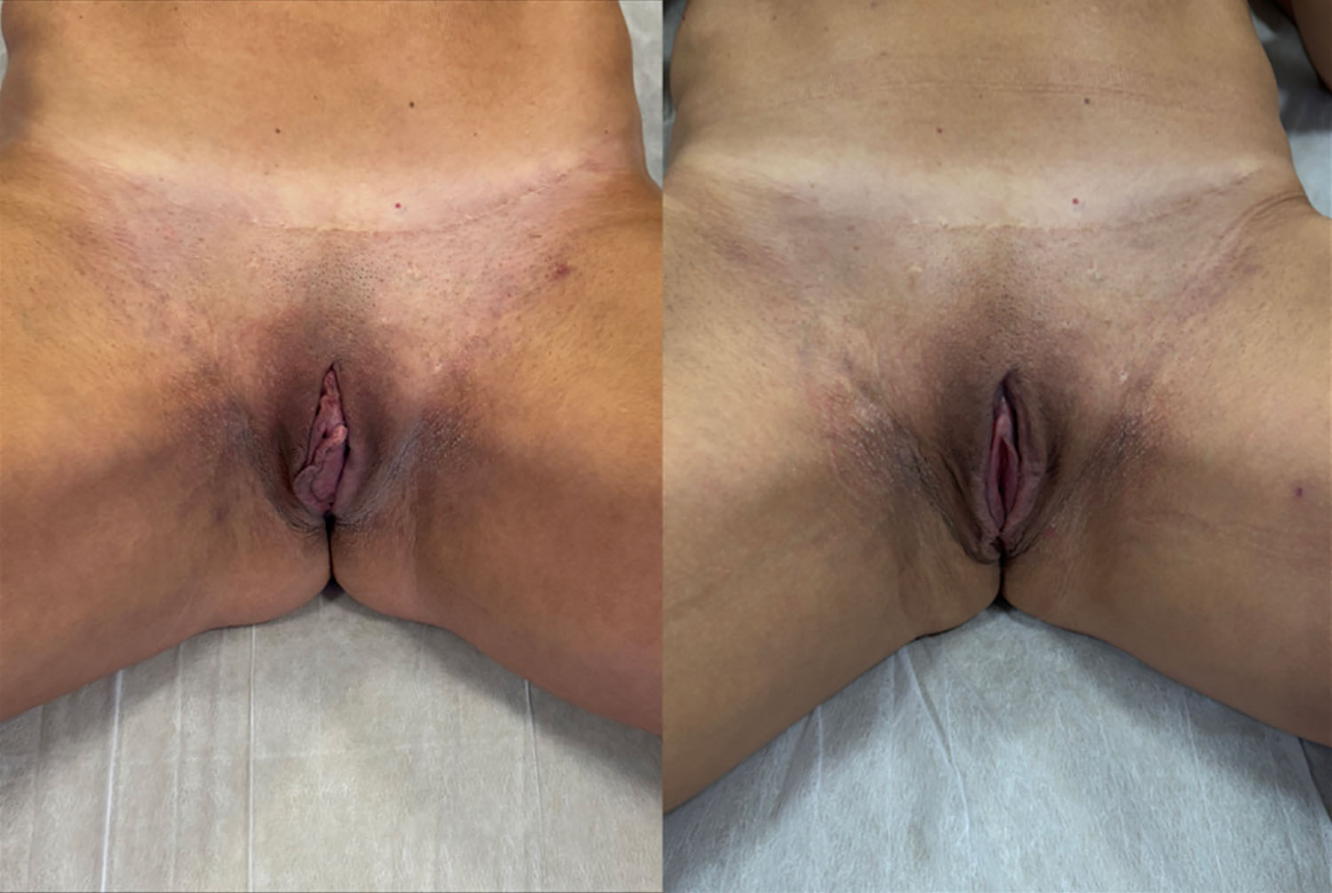
Pre-and postoperative lithotomy at 45º

**Fig 5:**
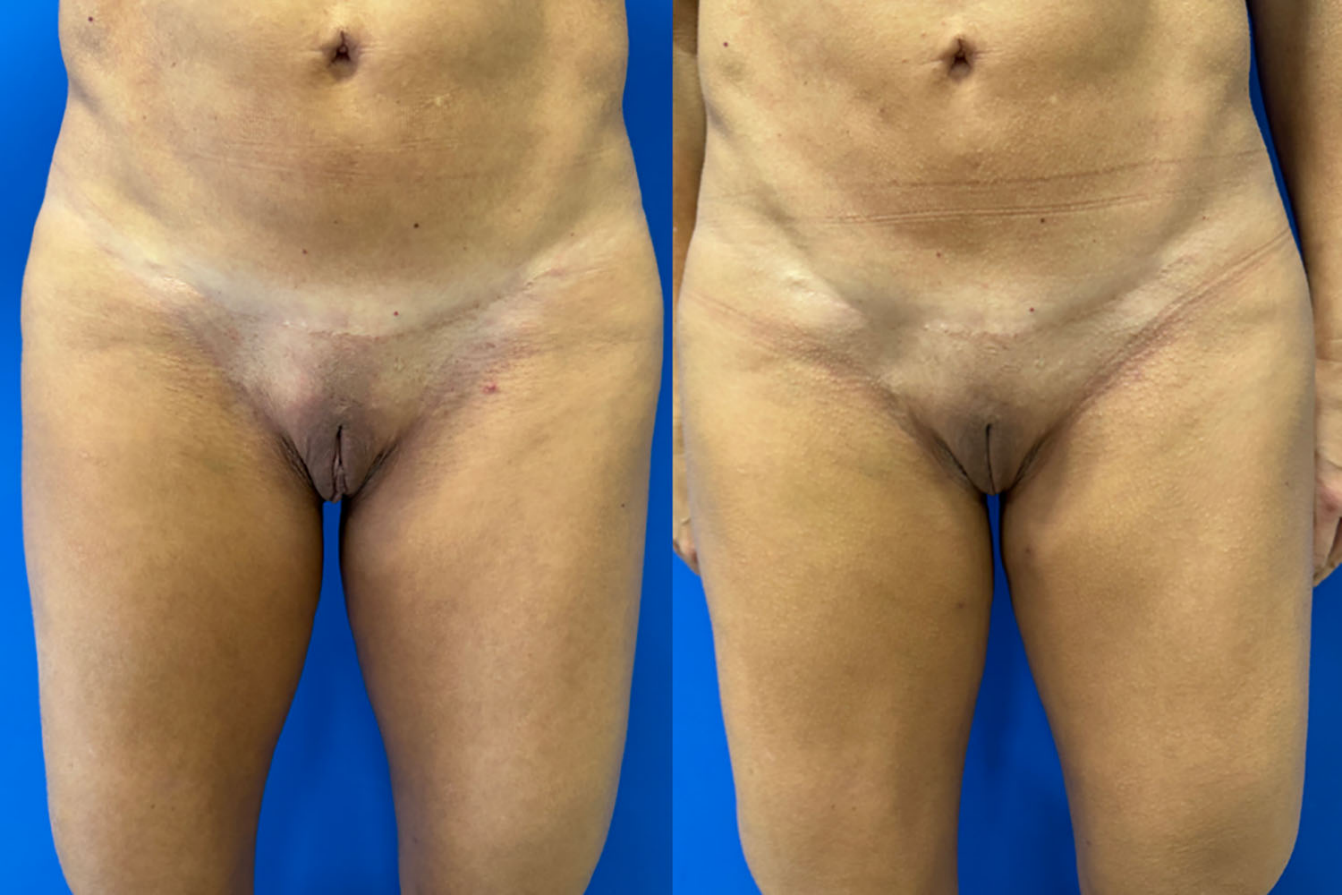
Pre-and postoperative frontal view

**Fig 6:**
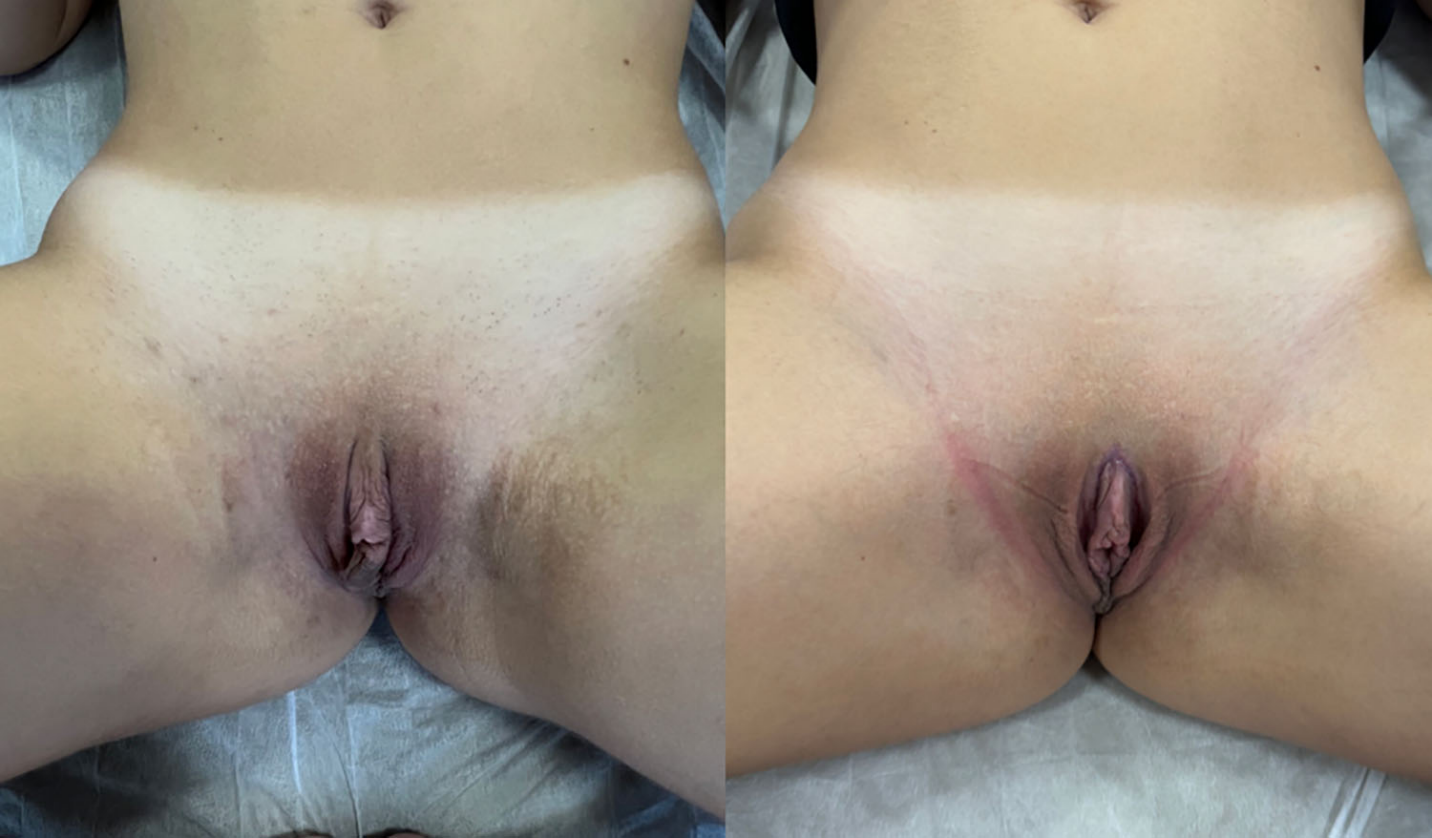
Pre-and postoperative lithotomy at 45º

**Fig 7:**
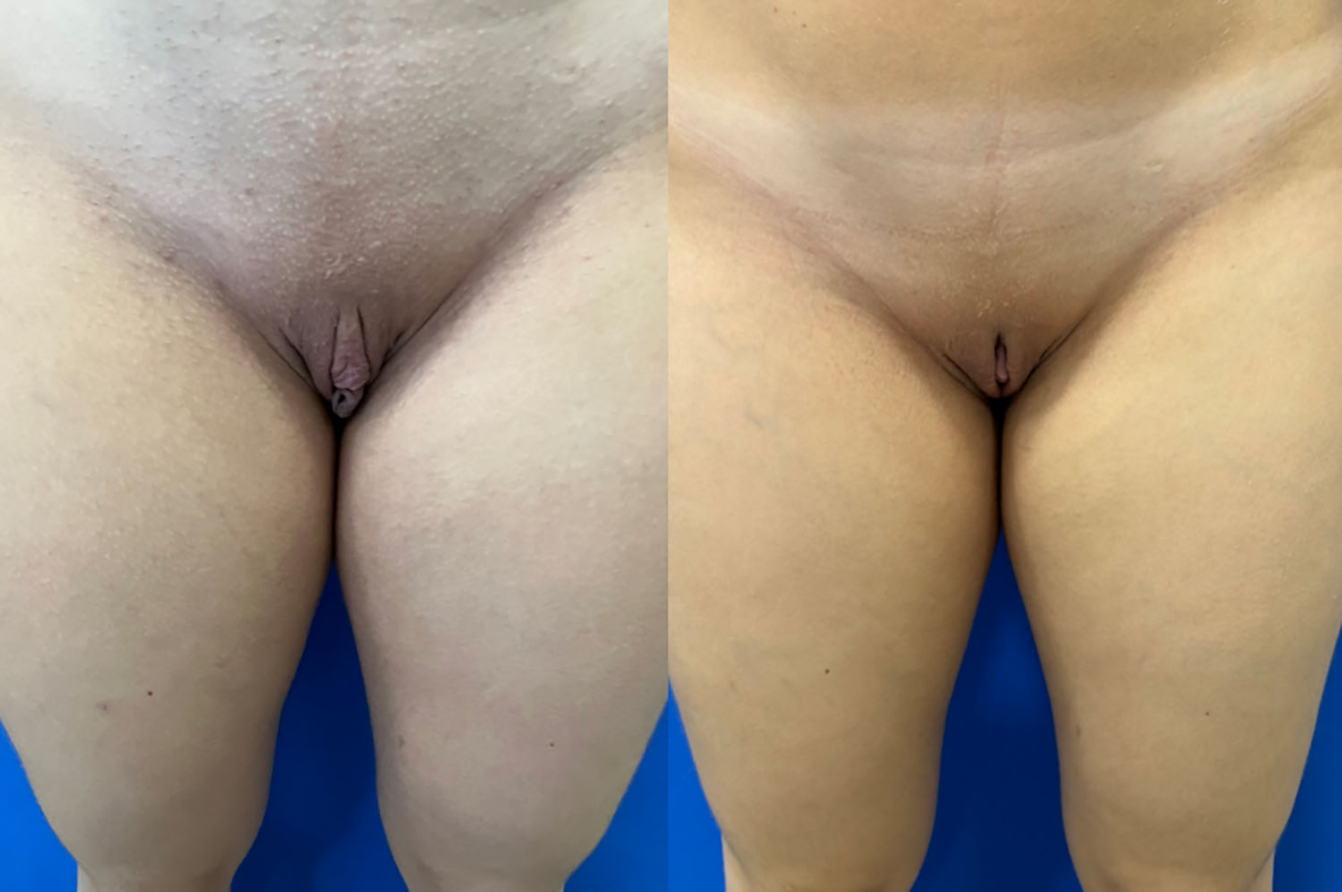
Pre-and postoperative frontal view

Figures 4, 5, 6, and 7 show the pre-and postoperative photographs of patients submitted to female genital plastic surgery "boomerang" technique [28].

**Fig 8:**
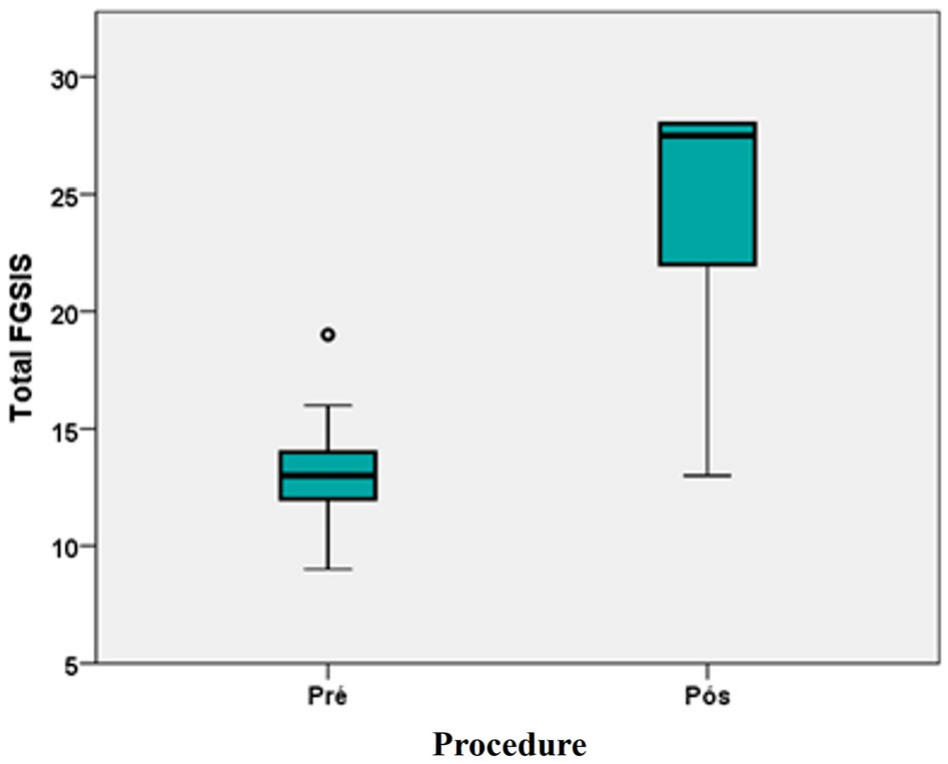
Box-plot of the total FGSIS in the pre-and postoperative of the sample, Brasília, DF, 2022

Figure 8 shows the total FGSIS score with a statistically significant increase (*p* < 0.05) compared to the preoperative and six postoperative month periods, demonstrating improvement in genital self-image. In the box-plot histogram, the preoperative presents a minimum score well below the minimum score stipulated for the postoperative period. The median also had an important variation from 13 to 27.5 compared to the preoperative and six postoperative month periods, as shown in Table 1.

For the SFQ-28 and FGSIS, 18 patients answered the questionnaire in the preoperative, 23 in the six postoperative month, and 10 in both periods (preoperative and six postoperative month). Table 3 shows that the domains of desire, arousal (cognitive), orgasm, enjoyment, and partner increased significantly (*p* < 0.05) after the surgery. Regarding arousal (sensation), arousal (lubrication), and pain, although there was clinical improvement in the six-month postoperative period, there was no significant improvement compared to the preoperative.

**Table 3:** Association of the preoperative and six-month postoperative values of the SFQ-28 of the patients submitted to the female genital plastic surgery, Brasília, DF, 2022

	Preoperative		Postoperative		p*
	Median	Interquatile range	Median	Interquatile range	
Desire	17.00	5.75	22.50	6.00	**0.044**
Arousal (sensation)	13.50	6.75	14.00	4.50	0.605
Arousal (lubrification)	8.00	3.00	9.00	2.50	0.395
Arousal (cognitive)	6.50	2.50	8.50	4.00	**0.020**
Orgasm	8.50	3.25	12.00	2.75	**0.008**
Pain	12.00	5.00	15.00	6.50	0.732
Enjoyment	17.50	9.25	24.00	4.00	**0.008**
Partner	8.00	4.25	10.00	0.50	**0.043**

*Wilcoxon test for related samples. SFQ-28: N = 10

Bold values indicate *p* < 0.05

Table 4 shows that all seven items of the FGSIS had a statistically significant improvement (*p* < 0.05) compared to the preoperative and six postoperative month period. Items 1 (feel), 2 (appearance), 4 (smell), and 5 (work) have an intrapersonal perspective, while items 3 (partner), 6 (healthcare), and 7 (shame) have an interpersonal perspective.

**Table 4:** Association of the preoperative and six-month postoperative values of the FGSIS of the patients submitted to female genital plastic surgery, Brasília, DF, 2022

	Preoperative		Postoperative		p*
	Median	Interquatile range	Median	Interquatile range	
1. Feel	1.00	1.00	4.00	1.00	< 0.001
2. Appearance	1.00	1.00	4.00	1.00	< 0.001
3. Partner	1.00	1.00	4.00	1.00	< 0.001
4. Smell	3.00	1.25	4.00	1.00	0.003
5. Work	2.00	1.00	4.00	1.00	0.002
6. Healthcare	2.50	1.25	4.00	0.25	0.001
7. Shame	1.00	1.00	4.00	1.00	< 0.001
Total FGSIS	13.00	2.50	27.50	6.25	< 0.001

*Wilcoxon test for related samples. FGSIS: N = 18

Table 5 shows no significant difference (*p* > 0.05) among the domains evaluated in the SFQ-28 and FGSIS comparing the place where the procedure was performed (SUS or the private clinic). All domains presented very close median values, even though they were patients with different socioeconomic levels.

**Table 5:** Association between the place where the procedure was performed (SUS or the private clinic) and the six-month postoperative values for the SFQ-28 and FGSIS of the patients submitted to female genital plastic surgery, Brasília, DF, 2022

	SUS		Private clinic		p*
	Median	Interquatile range	Median	Interquatile range	
Desire	20.50	7.00	22.00	6.00	0.322
Arousal (sensation)	12.50	6.00	15.00	9.00	0.110
Arousal (lubrification)	7.00	3.00	9.00	2.00	0.079
Arousal (cognitive)	6.50	4.00	8.00	5.00	0.971
Orgasm	12.00	5.00	11.00	3.00	1.000
Pain	15.00	1.00	12.00	8.00	0.079
Enjoyment	24.00	6.00	24.00	7.00	0.856
Partner	10.00	1.00	9.00	2.00	0.287
Total FGSIS	26.50	7.00	28.00	1.00	0.357

*Mann-Whitney U test for independent samples

The nonparametric correlation coefficient Spearman’s test was used to evaluate the correlation of coefficients of sexual function in the preoperative.

Table 6 shows that among the domains evaluated in the SFQ-28 and FGSIS, patients who answered higher desire values also did for arousal (cognitive), orgasm, pain, enjoyment, and partner in the preoperative. Higher arousal (sensation) values were correlated with higher orgasm values, and higher arousal (lubrication) values were associated with higher arousal (cognitive) and orgasm values. In the preoperative, arousal (cognitive) values were positively correlated with orgasm and enjoyment values; higher orgasm values were associated with higher enjoyment values, and higher enjoyment values were associated with higher partner values.

**Table 6:** Correlation analysis between the SFQ-28 and FGSIS in the preoperative period, Brasília, DF, 2022

Spearman’s test		Arousal (sensation) pre	Arousal (lubrification) pre	Arousal (cognitive) pre	Orgasm pre	Pain pre	Enjoyment pre	Partner pre	FGSIS pré
Desire pre	Coefficient	0.349	0.487	0.870	0.549	0.546	0.758	0.707	0.100
	*p*	0.221	0.077	< 0.001	0.042	0.043	0.002	0.005	0.734
	n	14	14	14	14	14	14	14	14
Arousal (sensation) pre	Coefficient		0.266	0.444	0.547	− 0.262	0.440	− 0.016	− 0.224
	*p*		0.358	0.112	0.043	0.365	0.116	0.958	0.441
	n		14	14	14	14	14	14	14
Arousal (lubrification) pre	Coefficient			0.635	0.679	0.194	0.469	0.437	0.345
	*p*			0.015	0.008	0.506	0.090	0.119	0.227
	n			14	14	14	14	14	14
Arousal (cognitive) pre	Coefficient				0.728	0.473	0.836	0.528	0.276
	*p*				0.003	0.088	< 0001	0.053	0.340
	n				14	14	14	14	14
Orgasm pre	Coefficient					0.341	0.818	0.257	0.172
	*p*					0.233	< 0.001	0.374	0.556
	n					14	14	14	14
Pain pre	Coefficient						0.467	0.510	0.011
	*p*						0.093	0.063	0.969
	n						14	14	14
Enjoyment pre	Coefficient							0.553	0.329
	*p*							0.040	0.251
	n							14	14
Partner pre	Coefficient								0.108
	*p*								0.712
	n								14

The SFQ-28 domains were classified regarding the presence or absence of sexual dysfunction. Table 6 shows for all domains, there was a decrease in patients with sexual dysfunction after genital plastic surgery. The McNemar statistical test was used to evaluate changes in nominal qualitative variables [29], which showed a statistically significant change only for orgasm, with a significant decrease in sexual dysfunction. For this domain, nine patients had sexual dysfunction in the preoperative, and only three maintained sexual dysfunction in the postoperative period (*p* = 0.031).

## Discussion

The improvement of sexual function in patients submitted to Female external genital plastic surgery (vulvoplasty) has been described since 2010. Goodman et al. showed that 64.7% improved sexual pleasure [30]. Veale et al. showed improvement in genital appearance and sexual satisfaction in women submitted to nymphoplasty [10].

According to Turini et al., women submitted to nymphoplasty showed improvement in the SFQ-28 in the pain item compared to the preoperative and six postoperative month periods [13]. In this study, there was a significant improvement in "desire", "arousal (cognitive)", "orgasm", "enjoyment" and "partner", compared in the preoperative and six postoperative month periods.

This difference in results may be associated with the difference in the surgical technique used in the two studies since, in the first, there was labia minora and excess clitoral hood resection only in the region lateral to the clitoris, as demonstrated, initially, by Alter while, in the current study, there was, in most patients (91.4%), labia minora resection associated with clitoral hood resection in the cephalic region, in addition to its repositioning [31]. That is, there was an expansion of the surgical procedure for the hood and clitoris [32]. Another important result is a significant improvement when sexual dysfunction was studied for the orgasm domain.

Regarding sexuality, women face a series of restrictions arising from the various cultural, religious, and moral factors defined by societies that misconstruct the healthy experience of sex. It is necessary to recognize sexual activity as an important element of human existence, maintaining emotional balance and the interaction between the woman, her body, and her partner [33].

There was a significant improvement in the genital self-image of women submitted to Female external genital plastic surgery (vulvoplastia)compared to the preoperative and six postoperative month periods, both in the total score and the seven items evaluated separately.

Some studies have shown an improvement in genital self-image after surgery, both retrospectively [34] and prospectively [8, 23], using the satisfaction questionnaire [24, 35] and the Genital Appearance questionnaire, even after two years of surgery [8].

Berman et al. elucidated the relationship between self-image as a component of sexual health in 2003 [25]. For Pujols et al., a positive relationship exists between sexual functioning, satisfaction, and all body image variables [26] Kloer et al. demonstrate that sexual health is multimodal for each person [27].

According to Vasconcelos et al., women with negative genital self-image were more likely to have sexual dysfunction, and young women with dissatisfaction with their bodies and with little or no sexual activity were more likely to present impairments in function and sexual satisfaction [5].

Gonçalves also showed that values ≥ 22 points in the FGSIS increased the chances of female sexual dysfunction [36].

Herbenick et al. demonstrated a positive relationship between genital self-image and sexual function. Women with a more positive self-image take more care of themselves and consult the gynecologist more frequently [3].

When women are dissatisfied with their genital appearance, some sexual disorders can occur. Consequently, the quality of life and relationship with partners is affected, which may precede conditions such as depression and low self-esteem [37].

This questionnaire FGSIS is widely used internationally and cover the self-image of the vulva well.

The fat graft in this region greatly benefits self-image, even when analyzed in isolation, as shown in the Cihantimur et al. study, which also improves firmness of the vaginal mucosa [38].

Our study has limitations, including not assessing urogynecological issues such as pelvic floor disorders, the lack of personnel resources to help some patients (illiterate) to answer the questionnaires. The small sample, although the results showed significant changes with important results, and 85.4% of the patients underwent the same type of surgery (labia minora resection, field resection plus clitoripexy), all of which were performed by the same surgeon.

## Conclusion

In our cohort, female external genital plastic surgery (vulvoplasty) elicited a positive relationship between genital self-image and sexual function, in addition to improving orgasm. Therefore, it is a consolidated surgical procedure with a low complication rate and self-satisfaction index.

## Data Availability

The authors confirm that the data supporting the findings of this study are available within the article. Furthermore, the data sets used and/or analyzed during the current study are available from the corresponding author upon reasonable request.
